# Insights into diagnostic difficulties in spinal muscular atrophy: a Case Report series

**DOI:** 10.3389/fgene.2024.1502444

**Published:** 2024-12-10

**Authors:** Kakha Bregvadze, Luka Abashishvili, Nana Nino Tatishvili, Teona Shatirishvili, Ana Bedoshvili, Gocha Chikvinidze, Arndt Rolfs, Volha Skrahina, Tinatin Tkemaladze

**Affiliations:** ^1^ Department of Molecular and Medical Genetics, Tbilisi State Medical University, Tbilisi, Georgia; ^2^ Neuroscience Department, M. Iashvili Children’s Central Hospital, Tbilisi, Georgia; ^3^ Department of Neurology, David Tvildiani Medical University, Tbilisi, Georgia; ^4^ Neurodevelopment Center, Tbilisi, Georgia; ^5^ Department of Neurology, I. Tsitsishvili Children’s New Clinic, Tbilisi, Georgia; ^6^ Medical Faculty, University of Rostock, Rostock, Germany; ^7^ Rolfs Consulting und Verwaltungs-GmbH (RCV), Berlin, Germany; ^8^ Department of Pediatrics, Givi Zhvania Pediatric Academic Clinic, Tbilisi State Medical University, Tbilisi, Georgia

**Keywords:** spinal muscular atrophy, SMA, *SMN1*, *SMN2*, Down syndrome

## Abstract

Spinal muscular atrophy (SMA) is a progressive neuromuscular disorder caused by mutations in *SMN1*, with disease severity influenced by the number of *SMN2* copies. Although SMA is one of the most common autosomal recessive disorders, molecular diagnosis still presents challenges. We present a case series illustrating the variable clinical presentations and diagnostic complexities of spinal muscular atrophy (SMA). Case 1 highlights the importance of multiplex ligation-dependent probe amplification (MLPA) and sequencing for detecting heterozygous deletions and novel variants. Case 2 highlights the limitations of neonatal screening, in which a heterozygous deletion was overlooked. Case 3 demonstrates the need for thorough clinical examination and relevant genetic testing in patients with dual diagnoses, in this case Down syndrome and SMA. In cases 4, 5, and 6, the pseudodominant inheritance pattern is examined in a familial context, highlighting the need for thorough genetic analysis. The presented case series emphasizes the diagnostic challenges and the crucial role of various molecular techniques in the accurate diagnosis and management of SMA.

## Background

Spinal muscular atrophies (SMAs) encompass a range of inherited neuromuscular disorders predominantly characterized by the degeneration of alpha motoneurons in the anterior horns of the spinal cord and brainstem (Mercuri et al., 2018). The most common form of SMA results from a defect in the survival motor neuron 1 (*SMN1*) gene located on 5q11.2-q13.3, also called 5q-SMA with an incidence of approximately 1:10,000 among newborns and a carrier frequency of 1:50 worldwide (Eggermann et al., 2020). Clinical manifestations are hypotonia, proximal muscle weakness and progressive muscle atrophy. Based on maximum motor function achieved and age of onset of the disease, SMA patients were originally classified into types 0, 1, 2, 3, and 4 (Munsat and Davies, 1992). A novel classification better reflects the continuum spectrum of the disease and divides patients into non-sitters (type 1), sitters (type 2 and 3 with loss of ambulation), and walkers (type 3 and 4 with preserved ambulation) (Mercuri et al., 2018).

*SMN* consists of nine exons with a stop codon near the end of exon 7. Two inverted *SMN* copies are present: the telomeric or *SMN1* and the centromeric or *SMN2* (Prior et al., 2011). *SMN1* and *SMN2* are located in a highly polymorphic 500 kb segment of a copy number variant (CNV) (Kleinle et al., 2023). Both *SMN1* and *SMN2* are nearly identical, with only five base pair differences (Lefebvre et al., 1995). *SMN1* produces correct RNA and fully functional protein, while *SMN2* mainly produces aberrantly spliced RNA and unstable protein. 5q-SMA patients lack *SMN1* and carry between one and six *SMN2* copies per genome. The number of *SMN2* copies influences the severity of the phenotype–patients with milder phenotypes were often found to have more *SMN2* copies (Wirth, 2000). In almost 96% of cases, 5q-SMA is caused by homozygous deletions of exons 7 and 8 or only of exon 7. The remaining 4% of affected individuals are compound heterozygotes with a *SMN1* deletion CNV on one allele and a pathogenic *SMN1* single nucleotide variant (SNV) on the second allele (Kleinle et al., 2023).

Although SMA is one of the most common autosomal recessive conditions, molecular diagnosis remains challenging. In newborn screening programs (NBS) currently testing for SMA, real-time PCR (qPCR) is used as a first-tier method to detect a homozygous deletion of exon 7 in *SMN1*. Positive NBS results must be confirmed by multiplex ligation-dependent probe amplification (MLPA), which can detect both homozygous and heterozygous deletions. Standard diagnostic testing in clinically suspected SMA is based on the detection of a homozygous exon 7 *SMN1* deletion by MLPA. If a clinically suspected SMA patient has only a single copy of *SMN1*, it is likely that the remaining copy contains an SNV, which requires gene sequencing (Prior et al., 2011; Wirth et al., 2020).

Here, we describe a case series that illustrates the different clinical scenarios highlighting the complex nature of spinal muscular atrophy (SMA) and the application of various diagnostic techniques that led to the final diagnosis of SMA.

## Case series

### Case 1

The proband was an 18-year-old male, the third child of healthy, unrelated parents. He was born in the 40th weeks of pregnancy with a birth weight of 3,600 g (53rd percentile), and height of 50 cm (<1 percentile). At 2 months, he was able to hold his head, at 6 months he was able to sit independently and at 13 months he was able to walk independently. At the age of 14 years, he developed progressive muscle weakness in his legs, difficulty in rising from a sitting position and in squatting, and difficulty in climbing stairs. He was not found to be fatigued when walking. Cognitive development was age-appropriate. Knee reflexes were not elicited.

Electromyography (EMG) testing showed neurogenic changes. Cardiac ultrasound was normal. Creatine kinase (CK) level were 440 U/L (N= <295 U/L) at the age of 14 years. Based on clinical findings and considering the broad spectrum of neuromuscular disorders, exome sequencing (ES) (Centogene, Germany) was performed and remained negative. Due to the limited capabilities of ES in detecting deletions associated with 5q-SMA, MLPA analysis (Centogene, Germany) of *SMN1* and *SMN2* was performed. A heterozygous pathogenic deletion encompassing exons 7 and *SMN1* and 2 copies of exons 7 and 8 of the *SMN2* gene were detected. As a next step, sequencing of the *SMN1* gene was performed (Centogene, Germany), which revealed a novel missense variant c.347T>C (p.Ile116Thr) in *SMN1* classified as likely pathogenic (PM2, PM5, PM1, PP2) (Richards et al., 2015). Based on clinical evaluation and genetic studies 5q-SMA was diagnosed.

The patient is currently being treated with risdiplam. During the 3-year treatment period, the patient was examined four times at 9-month intervals using the revised Hammersmith scale (RHS) for spinal muscular atrophy. The results were as follows: 47, 51, 55, and 57 respectively.

### Case 2

The proband was a 5-month-old girl, the second child to healthy unrelated parents. She was born by caesarean section at 35 weeks of gestation and weighed 2,700 g (55th percentile), and was 47 cm (52nd percentile). The patient required ventilatory support for 21 days, which eventually resolved. She was discharged home after 1 month. At 1.5 months of age, the patient presented with paradoxical breathing pattern and progressive hypotonia. At 5 months of age, she was admitted to the hospital due to worsening respiratory symptoms. Family history included an older brother who presented with similar symptoms and died at 6 months of age. His NBS result for SMA using real-time PCR was negative. The physical examination at 5 months of age revealed tongue fasciculations, muscle hypotonia, “frog-leg” posture, weak cry and breathing difficulties. The CK value at 5 months was 116 U/L (N= <295 U/L). The newborn screening for metabolic disorders was negative. A neuromuscular gene panel testing (including sequencing and deletion/duplication analysis) (Invitae, United States) revealed a pathogenic deletion (entire coding sequence) in *SMN1* and a nonsense variant c.549del (p.Lys184Serfs*29) in the *SMN1* classified as pathogenic (Richards et al., 2015). In addition, two copies of *SMN2* exon 7 were detected. Based on the clinical evaluation and genetic testing results 5q-SMA was diagnosed. She was intubated and required mechanical ventilation at the age of 9 months of age and died of ventilator-associated pneumonia at 11 months of age.

### Case 3

The proband was a 3-year-old boy, the second child of healthy unrelated parents. He was born at 37 weeks of gestation with a birth weight of 3,000 g (50th percentile), and a height of 51 cm (50th percentile). A few days after birth, he developed cyanosis while breastfeeding and an atrial septal defect was detected. At 2 days of age, he was admitted to the hospital and intubated. Physical examination revealed upward slanting eyes, round face, low nasal bridge, single transverse palmar crease, fifth digit clinodactyly, widely spaced toes, and paradoxical breathing pattern. Neurological examination showed diffuse hypotonia and a weak cry. The differential diagnosis included Down syndrome and SMA, so both the karyotype and the neuromuscular gene panel was performed. The G-banded karyotype from blood lymphocytes (550-bands) showed male karyotype 47,XY,+21 consistent with trisomic form of Down syndrome. The neuromuscular gene panel (including sequencing and deletion/duplication analysis) (Invitae, United States) revealed a pathogenic homozygous deletion of entire coding sequence of the *SMN1* gene and presence of 2 copies of the *SMN2*. Based on clinical evaluation and genetic studies, a dual diagnosis of Down syndrome and 5q-SMA were made.

Currently, the patient is on risdiplam and continues to require mechanical ventilation. There is minimal spontaneous movement in the distal extremities and contractures in knees and ankles. He requires a nasogastric tube feeding. The patient is conscious and responsive. Before starting treatment, the score of the Children’s Hospital of Philadelphia Infant Test of Neuromuscular Disorders (CHOP INTEND) scale was 3 points. The patient was evaluated three times during the 2-year treatment period and the score remained unchanged.

### Case 4, 5, and 6

The proband was a 45-year-old male who has been complaining primarily of muscle weakness, difficulty walking, climbing stairs, standing up from a seated position, and periodic muscle cramps for the past 30 years. The neurological examination revealed normal cognitive ability. Cranial nerve function was preserved. There was symmetrical, bilateral weakness of proximal muscles. The medical research council (MRC) scale for distal muscle groups was rated at 3 in both extremities, while the sensory and deep tendon reflexes were normal. Vision and hearing were normal.

Family history showed that the 24-year-old daughter and 22-year-old son had been affected in a similar way for about 10–12 years. Mother was healthy.

**FIGURE 1:**
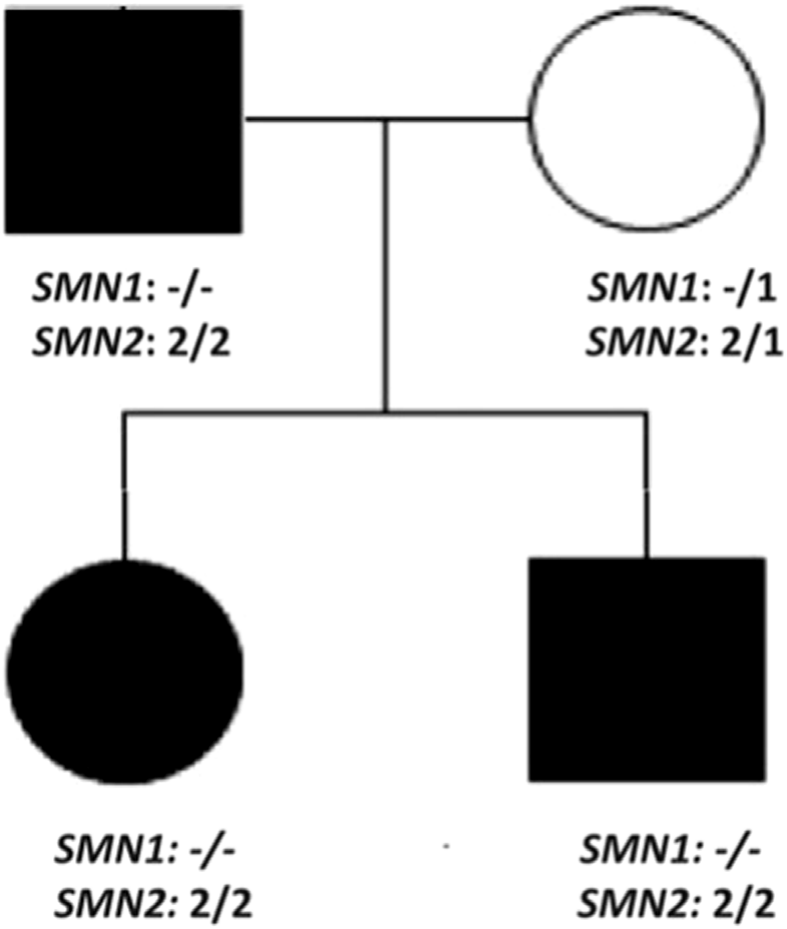
Pedigree of case 4, 5, and 6 demonstrating pseudodominant inheritance pattern. All three family members have homozygous pathogenic deletion of *SMN1* and 4 copies of *SMN2*.

EMG confirmed chronic denervation. CK was mildly elevated in all three affected family members. Autosomal dominant neuromuscular disease was suspected and ES (Centogene, Germany) of the entire family was performed which was negative. After 12 months we received an updated report for the entire family stating that during routine quality checks, by Next-generation sequencing (NGS)-based CNV analysis a variant was found in the *SMN1*. Deletion/duplication analysis was activated and MLPA analysis confirmed homozygous pathogenic deletion encompassing exon 7 of the *SMN1* and presence of 4 copies of *SMN2* in all affected family members. The mother had heterozygous deletion of *SMN1* and 3 copies of *SMN2* (Figure 1).

Currently, all three family members are treated with risdiplam. Before starting the treatment, the scores on were as follows: 52 for the father, 55 for the sister, 54 for the brother. During the 3-year treatment period, the patients were examined four times with at 9-months intervals and the scores remained unchanged.

## Discussion

With the introduction of approved molecular treatments for SMA, this disease has gained significant therapeutic importance in the field of neuromuscular disorders. SMA has been included in NBS in many countries world-wide as the treatment is the most successful when provided as early as possible. NBS is based on qPCR techniques, a rapid and cost-effective method suitable for initial screening, followed by confirmatory testing using MLPA. However, qPCR is not validated to identify heterozygous deletions, which are present in approximately 4% of patients. These individuals may be missed by screening and are likely to remain undiagnosed until clinical symptoms manifest. MLPA detects both homozygous and heterozygous *SMN1* deletions, however it not suitable for the detection of point mutations (Prior et al., 2011; Wirth et al., 2020). Therefore, sequencing of *SMN1* would need to be performed in a person with clinically suspected SMA. Due to the high homologies within the *SMN1*/*SMN2* locus, examination of the *SMN1* gene using next-generation short-read sequencing (srNGS) methods is not possible. Such regions of the genome are “dead zones” for NGS technology (Kleinle et al., 2023).

In case 1, the ES was negative due to the limitation of the technique and detection of a heterozygous *SMN1* deletion was possible only by MLPA. Further sequencing of *SMN1* confirmed diagnosis of SMA by identifying a novel heterozygous missense variant c.347T>C (p.Ile116Thr) in the *SMN1*. In case 2, the brother of the proband was not detected due to the limitation of qPCR to detect heterozygous deletion. In the proband, a gene panel testing revealed a compound heterozygous deletion and a nonsense variant c.549del (p.Lys184Serfs*29) in *SMN1*. Although both patients had two copies of *SMN2*, the clinical severity was very different. This variation can most likely be explained by the nature of the mutations. In case 1, the missense variant c.347T>C (p.Ile116Thr) most likely produces a protein with some residual function, resulting in a milder phenotype. In contrast, the nonsense variant c.549del (p.Lys184Serfs*29) in case 2 would result in a complete loss of protein function.

Case 3 highlights the importance of a thorough physical examination in diagnosing syndromic genetic conditions. Although the patient exhibited symptoms of central hypotonia and facial features consistent with DS, a detailed physical assessment also revealed prominent signs of peripheral hypotonia, suggestive of SMA. Both karyotyping and MLPA were performed, confirming the dual diagnosis of DS and SMA. The co-occurrence of DS and SMA is extremely rare, with only three cases reported in the literature. In one case, SMA was identified during the neonatal period in a child with already diagnosed DS and congenital hypothyroidism (Gulcan et al., 2005). Another report described a six-year-old child with DS who was diagnosed with SMA, with the delay in diagnosis likely attributed to overlapping symptoms (Tasende et al., 2022). The third case involved a 3-month-old girl presenting with features of both DS and SMA; chromosomal analysis confirmed the diagnosis of DS, while further investigations revealed a concurrent diagnosis of SMA (Gupta et al., 2024). Our case illustrates the importance of detailed clinical evaluation and application of relevant genetic investigations for the confirmation of dual diagnosis and the appropriate management and follow-up of patients.

In the familial case (patients 4, 5, and 6) the pedigree suggested an autosomal dominant inheritance pattern which influenced our decision to perform ES as a first-tier diagnostic test. Routine quality checks of the diagnostic lab was the key factor to initiate MLPA analysis which confirmed the diagnosis of SMA. Given the high carrier frequency of SMA, the possibility of pseudodominant inheritance in autosomal recessive diseases should be considered.

The presented case series illustrates the diagnostic challenges and the crucial role of comprehensive phenotyping and the application of relevant molecular techniques for the accurate diagnosis of SMA.

## Data Availability

The original contributions presented in the study are included in the article/supplementary material, further inquiries can be directed to the corresponding author.
